# Efficacy of surgical revision of mesh complications in prolapse and urinary incontinence surgery

**DOI:** 10.1007/s00192-020-04543-7

**Published:** 2020-10-09

**Authors:** Claudia R. Kowalik, Mariëlle M. E. Lakeman, Sandra E. Zwolsman, Jan-Paul W. R. Roovers

**Affiliations:** 1 Department of Obstetrics and Gynecology, Amsterdam University Medical Centre, Room H4-262, PO Box 22660, 1100 DD Amsterdam, The Netherlands; 2 Department of Obstetrics and Gynecology, BovenIJ ziekenhuis, Statenjachtstraat 1, Po box 37610, 1030 BD Amsterdam, The Netherlands

**Keywords:** Complications, Pelvic organ prolapse, Surgery

## Abstract

**Introduction and hypothesis:**

Women with mesh-related complications in prolapse (POP) and stress-urinary incontinence (SUI) surgery may benefit from operative mesh resection to alleviate symptoms. We hypothesized that mesh resection would alleviate symptoms and aimed to evaluate risks and benefits in these women.

**Methods:**

We carried out a cross-sectional study. Primary outcome was improvement specified as better, unchanged or worsened symptoms after mesh revision surgery. Secondary outcomes were health-related quality of life (HrQol) scores of validated questionnaires, surgical characteristics and physical findings at follow-up visits. Descriptive data were reported with mean and medians. Associations were calculated with Spearman correlation coefficient and chi-square test to determine statistical differences between groups.

**Results:**

Fifty-nine women who underwent mesh revision surgery between 2009 and 2016 were included. After a median follow-up of 1.7 (IQR: 1.1–2.4) years, 44 women (75%) reported improvement of symptoms. No significant surgical or patient characteristics were identified that could differentiate which patients did or did not experience cure or complications.A trend was observed to better HrQol scores in women who reported overall improvement after mesh revision surgery. Seventeen (29%) women needed a subsequent operation after mesh removal.

**Conclusions:**

This cross-sectional study shows that mesh revision surgery alleviates symptoms in 75% of women with mesh-related complications. Type of revision surgery and individual characteristics did not seem to matter to the individual chance of cure or complications. These data can facilitate the counseling of women considering mesh revision surgery.

## Introduction

Various surgical procedures for POP exist, but the perfect operation combining optimal cure rates and minimal morbidity has yet to be found.

The high failure rates of conventional surgery for POP resulted in the introduction of synthetic vaginal meshes [1]. The rationale for using these meshes is that they trigger fibroblasts to produce new collagen and elastin as part of the foreign body response they induce. Comparative studies have shown that the use of vaginal implants results in improved objective and subjective outcomes, although there are also studies that show no or limited benefit of the use of vaginal implants [2–5].

Synthetic mesh has also found its place in incontinence surgery. Since the 1990s polypropylene mesh slings have been inserted at the mid-urethral level to treat SUI with good results [6]. However, the use of vaginal implants for POP and SUI can result in specific complications such as mesh exposure (mesh protruding in the vagina), erosion (mesh protruding into the bladder or bowel) and pelvic pain.

The current literature mainly focuses on the incidence and severity of such mesh complications; however, management and improvement of mesh complications have previously been described. These studies report symptom relief and improvement varying between 51%–92% [7–9].

We analyzed the outcomes of mesh re-interventions in our tertiary referral center to document the risks and benefits and relate outcomes to the type of intervention and individual characteristics. These data can facilitate the counseling of women considering mesh revision surgery.

## Materials and methods

A cross-sectional study was performed at the Amsterdam University Medical Centers, location AMC in The Netherlands, with approval of the Medical Ethics Review Committee.

### Population

Patients were eligible for this study if they had a history of mesh revision surgery that had been performed in our tertiary referral hospital between 2009 and 2016. Eligible patients had a history of a transvaginal mesh (TVM) procedure, abdominal mesh procedure (sacrocolpo- or sacrohysteropexy; SCP) or mid-urethral sling surgery (MUS).

### Mesh types

Mesh types excised were: Perigee™, Apogee™, Elevate™, IVS™, Avaulta™, Prolift™, Gynemesh™, Gore-tex™, retropubic and transobturator midurethral slings.

In case of POP, mesh was categorized by the compartment (anterior, apical, posterior) of mesh implantation.

### Mesh revision

Mesh revision surgery was done under general or regional anesthesia. The operations were performed by an alternating team of three urogynecologists, with two uro-gynecologists operating together. We assessed which part or parts of the mesh were most likely causing the problem and needed to be addressed during surgery. We vaginally palpated the body of the mesh, the mesh arms and the connection of the mesh arms to the body. We recorded which parts were painful on examination, and these parts were removed or tension was released.

All women received prophylactic antibiotics and had an indwelling urinary catheter during the procedure. In all vaginal approaches, surgery commenced with hydro-dissection of the vaginal wall with adrenaline 1:200,000 combined with xylocaine 2%.

The surgical approach depended on the type of mesh complication or mesh type. We classified mesh revision surgery into four types of operations:

1. Removal of a locking eyelet or anchor (this is a polypropylene fixation ring respectively anchor utilized in the Elevate™ mesh kits): the anterior respectively posterior vaginal wall is incised, dependent on the type of mesh placed at the index surgery. After incision of the vaginal wall, the locking eyelet or anchor is identified, dissected and removed.
2. Exposure correction: the epithelium around the exposure is circumcised and mobilized. The vaginal epithelium surrounding the exposure is discarded. The exposed mesh is excised, and the vaginal epithelium surrounding the removed part of the mesh is mobilized and approximated by absorbable sutures.
3. Mesh resection/cleaving: Vaginal approach: the vaginal wall is incised and after identification of the mesh, it is dissected by keeping close proximity to the mesh, thereby preventing bladder damage or bowel injury. Tension on the mesh is released by cutting the mesh followed by resection, including the major part of the mesh arms.
  *Abdominal approach:* this can be the preferable route, by either laparoscopy or laparotomy, when removing mesh of SCP. It can be combined with a vaginal approach. The mesh is identified by careful dissection of the surrounding tissue and either completely removed or cut to release tension.
4. Removal of mesh from the bladder: this is performed by laparotomy and subsequent open cystotomy and excision of the exposed mesh from the bladder. After resection of the mesh the bladder mucosa is carefully examined to make sure that all mesh protruding from the bladder wall has been removed. If complete mesh resection from the bladder cannot be accomplished by cystotomy alone, the procedure is combined with a vaginal approach to achieve complete resection of the mesh erosion.

### Study procedures

Eligible patients received a letter regarding the study and could opt out if they did not want to be contacted. The patients that did not opt out were contacted by phone and asked to participate. Participants were asked to visit the study site. A gynecological examination was performed by a uro-gynecologist to assess POP by means of Pelvic Organ Prolapse Quantification (POP-Q). This examination was used to assess for POP recurrence. Recurrence was defined as stage 2 or more pelvic organ prolapse according to the POP-Q scoring system.

Data that could not be provided by the patient were abstracted from the medical records.

### Outcome measures

The primary outcome measure was perceived improvement after mesh revision surgery. This outcome was scored by asking patients to indicate whether they experienced improvement, no change or aggravation of their symptoms after revision surgery.

Subjective cure was assessed by the Patient Global Impression of Change (PGI-C) [10]. The PGI-C reports on outcome concerning activity, symptoms, emotions and general quality of life, related to the patient’s mesh complaints. Patients could select an answer on a 7-point Likert scale: “no change or worsening,” “almost no change,” “a little bit of improvement, but no notifiable change,” “a little bit of improvement, but no significant change,” “a little bit of improvement and a notifiable change,” better and a worthwhile change” and “very much better, a substantial change.” They were defined as cured when their answers to the PGI-C were “better” or “very much better.”

All patients were asked to complete the Urogenital Distress Inventory (UDI-6), Incontinence Impact Questionnaire (IIQ-7), Defecatory Distress Inventory (DDI) and Pelvic Organ Prolapse/Urinary Incontinence Sexual Function Questionnaire (PISQ-12) [11–15].

Serious adverse events (SAE) were categorized into per- and postoperative complications that required re-admittance to the hospital or repeat surgery.

### Statistical analysis

Data were analyzed using IBM SPSS Statistics 25.

Descriptive statistics were done as appropriate. For categorical data and not normally distributed numerical data, median and interquartile range (IQR) were reported. For continuous data, mean with standard deviation was reported. For frequencies, number plus percentage was given. Differences between groups were tested with independent t-tests for normally distributed data. In some cases only the year of mesh insertion was registered. In these cases the mesh insertion date was set to 1 January of that specific year to calculate the follow-up period.

Subjective improvement was scored in relation to the type of mesh revision operation that had been performed.

Scores for disease-specific questionnaires (UDI-6, IIQ-7, DDI, PISQ) were calculated appropriately [16]. HrQol scores were calculated and reported as an overall score and separately for women that experienced improvement, had experienced no change or had aggravation of symptoms after mesh revision surgery. Differences among these three groups were calculated with Kruskal-Wallis test for multiple comparisons of not normally distributed numerical data.

A chi-square test was done to assess statistical difference between the change in symptoms and the type of operation and to assess whether SAEs differed between types of surgery.

## Results

**Fig. 1:**
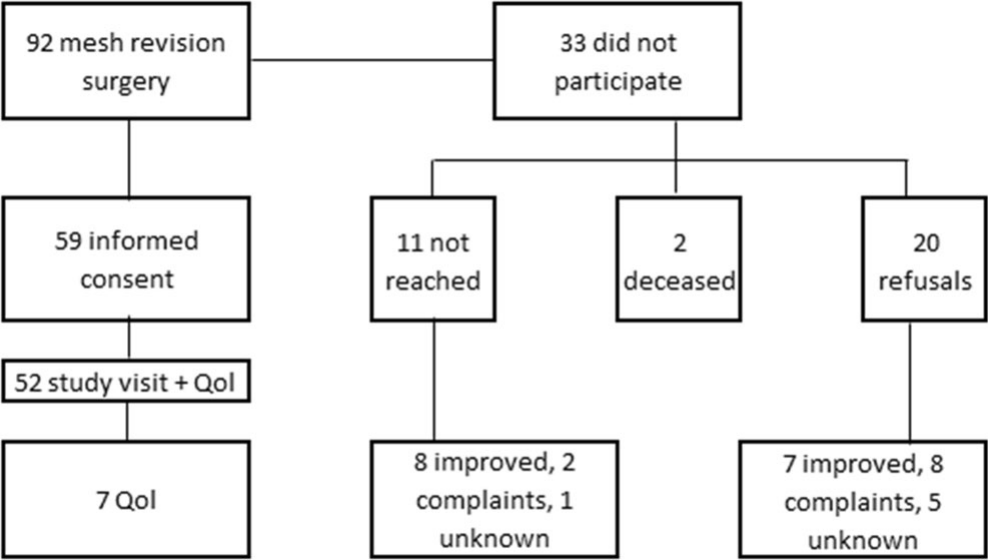
Flow chart of patients included in the study

Between 2009 and 2016, 92 patients had mesh revision surgery in our university hospital. Fifty-nine patients (64%) were included in this study. The women that were not included could not be reached or refused participation. The current status of their complaints were obtained by either chart review or telephone answers. This is shown in Fig. 1. Baseline characteristics are shown in Table 1. Nineteen women (32%) had persisting complaints after previous mesh removal surgery before they were referred to our center. The most important complaint as well as reason for opting for mesh revision surgery was pain (including dyspareunia). This was reported by 46 (78%) women.

**Table 1:** Baseline characteristics

Patient demographics	Missing	*n* = 59
Age (years) median (IQR)*		62 (54–67)
Follow-up (years) mesh insertion – intervention^¥^ median (IQR)	1	4.2 (1.2–6.9)
Time since mesh placement > 1 year *n* (%)	1	45 (77.6)
Follow-up (years) intervention- follow-up visit^¶^ median (IQR)		1.7 (1.1–2.4)
Postmenopausal *n, (%)*	12	36 (76.6)
BMI		25.7 (23.4–28.7)
Parity median (IQR)	1	2 (2–3)
Vaginal delivery *n* (%)	1	58 (98.3)
Cesarean section *n* (%)	1	4 (6.8)
Forceps/ventouse *n* (%)	9	16 (27.2)
Smoking *n* (%)		3 (5.1)
*History of prolapse surgery*		
Type of mesh *n* (%)		
Vaginal mesh implant		48 (80)
Midurethral sling (MUS)		6 (10)
Sacro-, colpo-/hysteropexia		6 (10)
Type of second mesh *n* (%)		
Vaginal mesh implant		8 (13)
Midurethral sling (MUS)		1 (1.6)
Sacro-, colpo-/hysteropexia		2 (3.3)
Type of third mesh *n* (%)		
Vaginal mesh implant		1 (1.6)
Midurethral sling (MUS)		0
Sacro-, colpo-/hysteropexia		1 (1.6)
Previous mesh revision surgery		19 (32)

^*^IQR: interquartile range

^¥^Time elapsed between the primary mesh insertion and the mesh revision under study

^¶^Time elapsed between the mesh revision under study and the follow-up visit

Chart review showed that fibrosis and too much tension of the mesh was the most frequent finding on physical examination and was observed in 36 (61%) women. Exposure was seen in 18 (31%) women. In three of these women both too much tension as well as an exposure was found.

Improvement was reported by 44 women (75%), 7 (13%) did not experience any change, and 3 (5%) experienced deterioration of their symptoms. In five subjects the outcome was missing. Subjective cure measured with the PGI-C was reported by 28 (47%) women (outcome missing in 4 patients). When comparing outcome per type of mesh revision surgery (MUS, abdominal mesh or vaginal mesh), there was no statistical difference in improvement or PGI-C scores between the various mesh categories.

Outcome per mesh type is shown in Table 2. Six women had a total mesh resection; in 39 women the mesh was partially resected. There was no significant difference in improvement between total and partial mesh resection (*p* = 0.52) or in serious adverse events (*p* = 0.94).

**Table 2:** Outcome categorized per mesh type

	*N*	Improved (*n*/%)	Similar (*n*/%)	Worsened (*n*/%)	*p* value	PGI-C (cure) (*n*/%)	*p* value
Vaginal mesh	47	34 (72)	7 (15)	4 (9)		22 (47)	
Abdominal mesh	6	5 (83)	0	1 (17)		4 (67)	
MUS	6	4 (67)	0	1 (17)		2 (33)	
Overall	59	44 (75)	7 (13)	3 (5)	0.41	28 (47)	0.38

The type of revision surgery as classified in the methods section did not show a statistical difference in the change in symptoms (*p* = 0.49).

The occurrence of a SAE was not related to the type of intervention (*p* = 0.74) or mesh type (MUS, vaginal or abdominal mesh) (*p* = 0.59).

No correlation was found between effects on symptoms after revision surgery and BMI, menopause, smoking, type of mesh graft, time between mesh insertion and moment of mesh revision, sexual activity and number of reoperations.

All vaginal mesh implants were removed vaginally. In one case an erosion of a retropubic midurethral sling has been removed abdominally because it had eroded into the bladder. In four cases the approach was abdominal and vaginal combined (twice by laparoscopy) and in one patient by abdominal approach only. These cases all concerned complications of an abdominal mesh.

In 41 (70%) women no concomitant vaginal surgery was performed during the mesh revision surgery; in 10 a native tissue repair and in 6 a MUS were executed simultaneously. In two women a mesh revision was combined with a vaginal mesh insertion. In one woman a mesh was inserted in the same compartment as the revision. In the other the mesh was inserted in a different compartment. There was no correlation regarding change of mesh-related symptoms and concomitant surgery (*p* = 0.82).

The surgical characteristics are shown in Table 3. SAEs were reported in eight patients. In one patient a bowel lesion occurred during abdominal mesh resection, and a jejunostomy had to be performed to manage the complication. In the vaginal mesh group, seven women had complications. Two women were registered as having a bladder lesion; in one of these women there was a minor suspicion of this lesion. One woman had a minor lesion of the serosa of the bowel. One woman had excessive bleeding during dissection of the anterior wall that was performed to insert an anterior mesh; during this operation the management of an exposure of a previous mesh did not cause the bleeding.

**Table 3:** Surgical characteristics of the study population

	Missing	*n* = 59
*Mesh categories revised*	1	
Single incision		15 (26.3)
Multiple incision		20 (35.1)
Mesh in more compartments		11 (18.6)
Sacrocolpopexia		6 (10.2)
MUS		6 (10.2)
*Mesh revisions performed*		
Exposure		7 (11.9)
Locking eyelet/anchor		5 (8.5)
Mesh resection		45 (76.3)
Mesh resection from bladder^*^		2 (3.4)
Time in surgery^*^ (minutes)	10	57 (36–103)
Blood loss^*^ (ml) (serious) adverse events	7	17.5 (0–50) 8 (13.3)
*Per operative complications*		5 (8.3)
Blood loss > 500 ml		1 (1.6)
Bladder lesion^¥^		2 (3.3)
Bowel lesion		2 (3.3)
*Postoperative complications requiring re- admittance/surgery*		3 (5.0)
Hematoma (hospital admittance)		1 (1.6)
Postoperative bleeding (repeat surgery)		2 (3.3)

Data are expressed as (1) median (*IQR = interquartile range) or (2) absolute numbers (percentage)

^¥^In 1 patient there was a minor suspicion of a bladder lesion, and an indwelling catheter was left in situ for 5 days

In 14 (24%) women, 23 reoperations were performed after the mesh revision surgery in our tertiary center. Indications for these reoperations were persistent mesh complications, POP surgery, SUI surgery or complication management. Some women needed a combination of procedures; therefore, the number of reoperations is higher than the number of patients. Ten women had a reoperation because of persisting mesh complications; one women needed two re-operations. Five women had subsequent POP surgery, and a MUS was placed in four women. Two women had a reoperation because of postoperative hemorrhage. At the follow-up visit, ten women were scheduled for a subsequent operation because of persisting mesh complications (*n* = 7), prolapse recurrence (*n* = 1) or urinary incontinence (*n* = 2). Of these ten women, seven already had a prior reoperation after the index mesh removal.

Overall POP recurrence in any compartment was seen in 18 women (31%; outcome missing in 12); these were mostly anterior and posterior compartment prolapses. Anterior compartment prolapse after anterior mesh revision occurred in five (8%) women. Two women who underwent an apical compartment mesh resection encountered prolapse recurrence of the apical compartment. Two woman had a posterior compartment recurrence after posterior mesh revision. None of the women had a prolapse POPQ stage 3 or 4. More than half of the women that had MUS revision surgery reported no to mild SUI symptoms.

Table 4 shows the HrQol scores of women who reported improvement, no change and worsened symptoms after mesh revision surgery. Women reporting improvement had better mean UDI-6 and IIQ-7 scores (not statistically significant).

**Table 4:** Health-related quality of life

	*n*	Improved	*n*	Similar	*n*	Worsened	*n*	*p*- value†
UDI-6^~^ mean (SD*)	46	34.5 ± 19.5	37	44.4 ± 25.3	6	66.7 ± 22.2	3	0.07
Irritative subscale	49	39.6 ± 22.9	40	41.7 ± 25.3	6	66.7 ± 33.3	3	0.34
Stress subscale	50	24.4 ± 21.1	41	41.7 ± 41.8	6	61.1 ± 34.7	3	0.10
Obstructive subscale	48	40.6 ± 28.3	39	50.0 ± 21.1	6	72.2 ± 9.6	3	0.10
IIQ-7^ǂ^ mean (SD*)	45	15.8 ± 20.2	38	22.5 ± 22.0	5	56.3 ± 14.7	2	0.09
Physical activity	47	20.8 ± 24.4	40	20.0 ± 29.8	5	66.7 ± 23.6	2	0.11
Mobility	48	17.1 ± 21.2	40	36.1 ± 37.1	6	58.3 ± 11.8	2	0.04
Social functioning	47	12.8 ± 19.7	39	33.3 ± 36.5	6	50.0 ± 23.6	2	0.04
Emotional health	47	17.1 ± 26.9	39	33.3 ± 31.6	6	50. ± 0.00	2	0.09
DDI** mean (SD*)	41	15.5 ± 13.9	36	6.1 ± 6.1	3	18.2 ± 17.1	2	0.41
Constipation	43	16.2 ± 22.0	37	5.6 ± 9.6	3	11.1 ± 19.2	3	0.65
Painful defecation	45	18.4 ± 26.2	39	11.1 ± 19.2	3	16.7 ± 28.9	3	0.92
Fecal incontinence	47	5.3 ± 10.8	41	5.6 ± 9.6	3	11.1 ± 19.2	3	0.80
Flatus incontinence	47	25.2 ± 28.6	41	11.1 ± 19.2	3	22.2 ± 38.5	3	0.70
PISQ-12^¥^ summary score	25	32.3 ± 3.6	23	30.5 ± 6.4	2		0	0.62

^*^SD: standard deviation

^~^UDI-6: Urogenital Distress Inventory

^ǂ^IIQ: Incontinence Impact Questionnaire

**DDI: Defecatory Distress Inventory

^¥^PISQ-12: Pelvic Organ Prolapse/Urinary Incontinence Sexual Function Questionnaire

^†^Significance cutoff at *p* < 0.05

Not all women answered all questions

## Discussion

This cross-sectional study demonstrates that 75% of women undergoing mesh revision surgery because of mesh-related complications after POP or SUI surgery experienced an improvement of symptoms, while in 5% symptoms worsened. Subjective cure measured with the PGI-C was reported by 47% of patients. There was no statistical difference in outcome among the MUS, abdominal and vaginal mesh resections. Twenty-nine percent of women were indicated to need an additional operation because of persistent mesh complications, POP recurrence or SUI.

Improvement of symptoms or the occurrence of SAEs was not related to the type of intervention performed as classified in the Methods section. This outcome was scored after a median follow-up of 1.7 (IQ range: 1.1–2.4) years after revision surgery.

The percentage of symptom relief is consistent with other studies. Reports of symptom relief vary between 46%–92% with the difference that these other reports have considerably shorter follow-ups than our study [8, 17, 18] except for the study of Warembourg et al., which report a cure rate of 78% with a mean follow-up of 41 months (95%CI: 34.3–47.7) [9].

The main reason for having mesh revision surgery in our population was pain (including) dyspareunia, which was reported by 78% of patients. Pain being the main indication for revision surgery has also been reported by other researchers [17, 19]. Unfortunately, pelvic and vaginal pain is difficult to treat. The causal factor of pain after mesh surgery remains unclear, but it has been hypothesized that too much tension on the mesh, fibrosis and exposure are factors that contribute to pain symptoms.

In the majority of women in our study (61%) fibrosis or too much tension on the mesh assessed by palpation was found at pelvic examination prior to revision surgery.

When women present to our clinic with pain complaints after a mesh insertion, we examine them and try to objectify whether their complaints are due to hypertonia of the pelvic floor muscles or because of a mesh complication. In case of the first, we refer women to pelvic floor physical therapy, but when the mesh itself seems to cause the problem we proceed to surgical resection. In some women additional pelvic floor physical therapy is needed after surgery.

Our surgical approach to the treatment of mesh complications is to release the tension and remove the most painful part of the mesh.

How much of the mesh needs to be removed when pain complaints are the indication for revision surgery depends on the severity of symptoms, location of tension/fibrosis and risk of complications due to proximity of the mesh to the bladder or rectum. Consequently, in some patients as much mesh as possible was removed, but in most mesh remnants were left in situ.

There was no statistical difference in change of symptoms among the four surgical approaches that we use in our hospital or category of mesh (MUS, abdominal mesh, vaginal mesh) that was removed or in improvement between total and partial mesh removal. It seems that resection of as much mesh as possible is not mandatory to achieve symptom relief. Wolff et al. also concluded in their review that total mesh removal is not always more beneficial to the patients compared to partial mesh resection [20].

This is important information to share with the patient, since some patients believe that only complete removal of the mesh will result in resolution of their symptoms.

When informing women about mesh revision surgery, the chance of having prolapse recurrence should be the subject of the counseling. Ideally, surgery should alleviate mesh-related complaints, without causing new prolapse-related problems. In this study we showed that the chance of prolapse recurrence in the specific compartment where the mesh had been removed was most common in the anterior compartment (8%). This anterior recurrence rate was less than in the study of Marcus Braun that reported 19% cystocele recurrence after removal of the vesico-vaginal mesh [21]. This might be explained by the fact that Marcus Braun et al. saw most recurrences after complete mesh resection, whereas most patients in our study underwent a partial resection.

In MUS revisions there is a risk of relapse of SUI symptoms. In our population, 33% had SUI at the follow-up visit, but one must consider that tape revision was only performed in six patients when interpreting this outcome. Other studies describe that 14–23% of women have surgery for recurrent SUI after tape revision and 49% have SUI recurrence [22–24].

HrQol was assessed at the follow-up visit in the current study. The UDI-6 and IIQ-7 scores were better in the women who reported improvement after revision, although this outcome did not reach statistical significance except for the subdomains of mobility and social functioning of the IIQ-7. However, one should consider that the sample sizes were small.

This study has some limitations that need to be addressed. Not all women that had mesh revision surgery in our tertiary center consented to participate in the study, and some women only consented to fill out the HrQol questionnaires. The results can be affected by selection bias.

In this study, various types of mesh have been revised. The outcome could differ depending on the type of mesh that has been revised, but on the contrary this study is strengthened by the fact that it gives a representation of the daily practice in a tertiary center. Our center has a special interest in mesh-related complications and has developed much experience in the treatment of these problems. These facts have to be kept in mind when interpreting the current data. The outcome of this study may not be generalizable to other settings.

Another strength of this study is that it reports on subjective outcomes after revision surgery, including the outcome of standardized questionnaires concerning HrQol after a long-term follow-up.

## Conclusions

This cross-sectional study shows that mesh revision surgery alleviates symptoms in 75% of women who had mesh-related complications after POP and/or SUI surgery. The type of revision surgery and individual characteristics did not seem to matter to the individual chance of cure or complications. Seventeen (29%) women needed a subsequent operation after mesh removal. These data can facilitate the counseling of women considering mesh revision surgery.
